# Conditional ATXN2L-Null in Adult Frontal Cortex CamK2a+ Neurons Does Not Cause Cell Death but Restricts Spontaneous Mobility and Affects the Alternative Splicing Pathway

**DOI:** 10.3390/cells14191532

**Published:** 2025-09-30

**Authors:** Jana Key, Luis-Enrique Almaguer-Mederos, Arvind Reddy Kandi, Meike Fellenz, Suzana Gispert, Gabriele Köpf, David Meierhofer, Thomas Deller, Georg Auburger

**Affiliations:** 1 Clinic of Neurology, Experimental Neurology, University Hospital, Goethe University Frankfurt, Heinrich-Hoffmann-Str. 7, 60528 Frankfurt am Main, Germany; jana.key88@gmail.com (J.K.); lalmaguermederos@gmail.com (L.-E.A.-M.); arviarvindreddy514@gmail.com (A.R.K.); gispert-sanchez@em.uni-frankfurt.de (S.G.);; 2 Institute for Clinical Neuroanatomy, Dr. Senckenberg Anatomy, Fachbereich Medizin, Goethe University Frankfurt, 60528 Frankfurt am Main, Germany; m.fellenz@em.uni-frankfurt.de (M.F.); t.deller@em.uni-frankfurt.de (T.D.); 3 Max Planck Institute for Molecular Genetics, Ihnestraße 63-73, 14195 Berlin, Germany; meierhof@molgen.mpg.de

**Keywords:** poly(A)-binding protein, ribonucleoproteins, stress granules, open field locomotion, label-free mass spectrometry, NAA38, NSUN2, RPS3, MRPL14, SRSF11

## Abstract

**Highlights:**

**What are the main findings?**

- Constitutive knock-out of ATXN2L across LSMAD and PAM2 is embryonically lethal, confirming its essential role in development.
- Conditional deletion of ATXN2L across LSMAD and PAM2 postnatally in cortical neurons reduces spontaneous movement and alters alternative splicing pathways.

**What are the implications of the main findings?**

- ATXN2L is indispensable for embryonic survival and neuronal function, highlighting its non-redundant role, in contrast to its paralog ATXN2.
- The LSMAD and PAM2 domains of ATXN2L likely impact nuclear splicing, despite the protein’s perinuclear localization.

**Abstract:**

The Ataxin-2-like (ATXN2L) protein is required to survive embryonic development, as documented in mice with the constitutive absence of the ATXN2L Lsm, LsmAD, and PAM2 domains due to knock-out (KO) of exons 5–8 with a frameshift. Its less abundant paralog, Ataxin-2 (ATXN2), has an extended N-terminus, where a polyglutamine domain is prone to expansions, mediating vulnerability to the polygenic adult motor neuron disease ALS (Amyotrophic Lateral Sclerosis) or causing the monogenic neurodegenerative processes of Spinocerebellar Ataxia Type 2 (SCA2), depending on larger mutation sizes. Here, we elucidated the physiological function of ATXN2L by deleting the LsmAD and PAM2 motifs via loxP-mediated KO of exons 10–17 with a frameshift. Crossing heterozygous floxed mice with constitutive Cre-deleter animals confirmed embryonic lethality among offspring. Crossing with CamK2a-CreERT2 mice and injecting tamoxifen for conditional deletion achieved chimeric ATXN2L absence in CamK2a-positive frontal cortex neurons and reduced spontaneous horizontal movement. Global proteome profiling of frontal cortex homogenate showed ATXN2L levels decreased to 75% and dysregulations enriched in the alternative splicing pathway. Nuclear proteins with Sm domains are critical to performing splicing; therefore, our data suggest that the Like-Sm (Lsm, LsmAD) domains in ATXN2L serve a role in splice regulation, despite their perinuclear location.

## 1. Introduction

The Sm domain is an ancient RNA-binding motif with oligo(U) specificity that assembles into heteroheptameric rings [1], with similarity to nuclear Sm core ribonucleoprotein rings in the spliceosome and to cytosolic Like-Sm (LSm) ribonucleoprotein rings that mediate mRNA decapping and decay [2]. In bacteria and archaea, the Sm domains in Hfq homologs have been extensively studied to characterize their best-known role in intron splicing from pre-mRNA and to demonstrate their functions as chaperones that mediate interactions between regulatory non-coding RNAs and their targets, as well as functions for the maturation of tRNAs/rRNAs [3].

With the evolution of multi-domain proteins in eukaryotic cells, RNA processing has become more efficient, particularly with the discovery of a cytoplasmic ribonucleoprotein family with a length of 600 to 1000 amino acids (exemplified by the *Saccharomyces cerevisiae* yeast protein Pbp1), which provides binding sites for (i) RNA in the Lsm domain, (ii) RNA helicases in the area from Lsm to Lsm-associated domain (LsmAD) sequences, and (iii) poly(A)-binding proteins at the PAM2 motif. In cellular stress periods, Pbp1 and all its orthologs relocalize away from the translation apparatus to stress granules, where RNA quality control and triage are performed [4,5,6,7]. Other currently studied family members include ATX-2 in *Caenorhabditis elegans* worms [8,9,10,11,12,13], dATX2 in *Drosophila melanogaster* flies [14,15,16,17,18,19,20,21,22,23,24,25,26,27,28], and ATXN2 in *Gallus gallus* birds [29]. With increasing organism mass, and outside the temperate sea environment, gene duplication has been conserved from ray-finned fish to mammals. The less abundant—but N-terminally much extended—version with around 1300 amino acids is called Ataxin-2 or hATXN2 in humans; conversely, a relatively unchanged and more abundant version with around 1000 amino acids is known as Ataxin-2-like or ATXN2L. Furthermore, land plants carry two gene copies (known in *Arabidopsis thaliana* as CID3/CID4) [30].

Research on patients with autosomal-dominant, chronically progressive spinocerebellar ataxia type 2 (SCA2) has identified unstable expansions in the polyglutamine (polyQ) domain, surrounded by Proline-rich motifs (PRM) in the N-terminus of hATXN2, as a cause of disease [31,32,33]. This has provided a name for this gene family and initial insights into its physiological and pathological roles [10,34,35,36,37,38,39,40,41,42,43,44,45,46,47,48,49,50,51,52,53]. Subsequent findings show that this gene modifies the risk and disease progression of adult motor neuron degenerations such as Amyotrophic Lateral Sclerosis (ALS) [54,55,56,57,58]. This has intensified analyses of its impact on RNA maturation and RNA quality control in stress granules [15,18,19,22,25,28,59,60,61,62,63,64,65,66,67,68,69,70]. Mammalian ATXN2 appears to mediate rapid stress responses and increase fitness, but its deletion results in obesity, hepatosteatosis, hyperinsulinemia, and hypercholesterolemia [39,48]; however, its excessive gain-of-function restricts nutrient endocytosis, represses mTORC1-dependent growth, decreases cholesterol biosynthesis, and depletes myelin lipids [41,50,66,71]. Interestingly, the precise circadian clock rhythms are regulated by oscillating waves of translation, where ATXN2 depletion triggers an advanced phase shift, whereas ATXN2L depletion triggers a delayed phase shift [68]; therefore, the function of these two ribonucleoproteins may be complementary or antagonistic.

Pathogenic mutations in patients have not been documented for ATXN2L, and only very few studies have been dedicated to mammalian ATXN2L. It conserves the classical domain combination Lsm-LsmAD-PAM2 but has a PRM and MPL binding region at its N-terminus [72] and an additional Pat1 homology sequence at its C-terminus (which is absent from ATXN2 [62]). Pat1 proteins usually interact with the cytosolic Lsm1-7 ring to promote deadenylation-dependent decapping and degradation of 3′ UTR AU-rich mRNAs in P-bodies [73,74,75,76]; however, they can undergo nuclear relocation to Cajal bodies/speckles upon stress and interact with nuclear Lsm2-8 rings complexed with U6 snRNA to promote splicing [77,78,79], with a prominent role in the growth of synaptic terminals [80]. ATXN2L can localize to nuclear splicing speckles [62], and its nuclear localization depends on its PRMT1-dependent arginine methylation [81]. Its normally cytosolic distribution shows more of a perinuclear concentration than ATXN2 [82]. Upon constitutive knock-out of *Atxn2l* exons 5–8 with a subsequent frameshift in the mice, their homozygous offspring suffered from mid-gestational embryonic lethality, with brain neuronal lamination defects and apoptosis [83]. In proteome profiles of murine embryonic fibroblasts, ATXN2L loss results in similarly strong deficits in RNA processing factor NUFIP2 and nuclear envelope factor SYNE2; both proteins are interactors of ATXN2L [82], but it remains unclear which protein dysregulations mediate the preferential ATXN2L impact on neural tissue.

To obtain an initial understanding of ATXN2L deficiency’s impact on adult neurons at the behavioral, cellular, and molecular levels, we generated the first mouse line with floxed *Atxn2l* for conditional manipulations, where an N-terminal fragment of ATXN2L with the Lsm domain may still exist, but the remaining protein is ablated.

## 2. Materials and Methods

### 2.1. Mouse Breeding and Genotyping

All animal experiments complied with the German Animal Welfare Act, European Directive 86/609/EWG (24 November 1986, Annex II), and the ETS123 guidelines (European Convention for the Protection of Vertebrate Animals). Mice were maintained in the Central Animal Facility (ZFE) of the Goethe University Medical School under standard conditions: individually ventilated cages containing nesting material, a 12 h light–12 h dark cycle, controlled temperature and humidity, and free access to food and water. Colony maintenance and expansion were achieved by breeding heterozygous carriers. For experimental comparisons, sex-matched mutant and wild-type (WT) littermates were housed together. Both male and female animals were included in all experiments. Ethical approval was obtained from the Regierungspräsidium Darmstadt (permit number V54-19c20/15-FK/2032). The analysis of Ataxin-2-like isoforms and the subsequent development of an *Atxn2l* conditional knock-out (cKO) mouse line in the C57BL/6 genetic background via homologous recombination were outsourced to the Genoway company (Lyon, France). The targeting vector was designed using sequences from *Mus musculus* strain C57BL/6J chromosome 7, GRCm39, NC_000073.7, nucleotide positions 126075978 to 126115977. Sperm from the successfully floxed *Atxn2l* mice were deposited at Genoway. SnapGene (Boston, MA, USA, Version 8.0.2) was used for structural analysis and primer generation.

To trigger the *Atxn2l*-KO constitutively, mice with targeted flox site insertion were bred with pan-Cre-deleter mice to obtain heterozygous *Atxn2l*-KO mice for intercrosses. To selectively trigger the *Atxn2l*-cKO in CamK2a-positive cells, homozygous *Atxn2l*-flox mice were crossed with CamK2a-Cre/ERT2 mice (B6;129S6-Tg(CamK2a-Cre/ERT2)1Aibs/J, Jackson Laboratories, Bar Harbor, Maine, USA). Resulting double mutants that were heterozygous for *Atxn2l*-flox and expressed Cre recombinase (*Atxn2l*-WT/flox, CamK2a-Cre/ERT2-Tg) were then crossed with homozygous *Atxn2l*-flox mice to generate homozygous *Atxn2l*-flox mice with transgenic Cre (Cre-Tg). Homozygous *Atxn2l*-flox mice without Cre served as littermate controls. All genotyping primers are provided in Table S1.

To genotype the floxed *Atxn2l*, DNA from ear tissues was extracted using the hot shot method [84], performing the PCR with 1 µL of DNA in a reaction mix with AmpliTaq (Thermo Fisher, Waltham, MA, USA). The PCR conditions were 3 min at 94 °C; 35 cycles of 30 s at 94 °C, 30 s at 65 °C, and 30 s at 72 °C; followed by 5 min at 72 °C. The expected bands were 315 bp for the WT allele and 405 bp for the floxed allele.

To genotype the Cre transgene, a touchdown PCR was performed, as recommended by the Jackson website (https://www.jax.org/Protocol?stockNumber=012362&protocolID=27167, last accessed on 6 June 2024). Then, 5 min at 94 °C was followed by 10 cycles with a decrease of 0.5 °C per cycle with 30 s at 94 °C, 30 s at 65 °C, and 30 s at 68 °C. This was followed by 28 cycles of 30 s at 94 °C, 30 s at 60 °C, and 30 s at 72 °C, with 7 min at 72 °C at the end. The expected band sizes were 521 bp for the control and 200 bp for the presence of Cre.

To genotype the *Atxn2l*-KO, the PCR conditions were 3 min at 94 °C; 35 cycles of 30 s at 94 °C, 30 s at 66 °C, and 30 s at 72 °C; followed by 5 min at 72 °C. The expected bands were 151 and 3808 bp for the WT allele and 276 bp for the KO allele.

### 2.2. Assessment of Embryonic Lethality in Constitutive Atxn2l-KO Mice

After excision of the neo cassette and the floxed region in vivo, 2 heterozygous mice with constitutive deletion of *Atxn2l* exons 10–17 were obtained, which were then crossed with C57BL/6 mice to generate a colony. Multiple breeding units of male and female heterozygotes were used to analyze the offspring for the viability of *Atxn2l*-KO mice and the distribution of heterozygous versus WT animals among the offspring. In total, 100 litter pups were produced and genotyped.

### 2.3. Immunohistochemistry

Serial frontal sections of 50 µm thickness were cut using a vibratome (Leica VT 1000 S Leica, Wetzler, Germany) and collected in PBS. Individual sections were incubated in a blocking solution (10% normal goat serum (NGS), 0.5% Triton X-100 in PBS) at RT for 2 h. Afterward, sections were incubated overnight at RT in primary antibodies (rabbit-anti-ATXN2L, 1:300, Proteintech Cat# 24822-1-AP, RRID: AB_2879743; mouse-anti-CAMK2a, 1:500, Santa Cruz Biotechnology Cat# sc-70492, RRID: AB_1119957) diluted in antibody solution (5% NGS, 0.2% Triton X-100 in PBS). Sections were washed three times (PBS, 10 min each) and then incubated in the dark for 2 h at room temperature with secondary antibodies diluted in antibody solution (goat-anti-rabbit Alexa Fluor 568, 1:1000, Thermo Fisher Scientific Cat# A-11036, RRID: AB_10563566; goat-anti-mouse Alexa Fluor 488, 1:1000, Thermo Fisher Scientific Cat# A-11029, RRID: AB_2534088). Sections were then washed three times in PBS for 10 min, with the second wash containing the nuclear stain Hoechst (1:5000). Finally, sections were mounted on glass slides with mounting medium (Dako/Agilent, Santa Clara, CA, USA).

### 2.4. Imaging

Fluorescent widefield overview images were acquired with an IXplore Live IX83 LED (Evident) using a 4× objective (UPlanXApo, NA 0.16, Thermo Scientific, Waltham, MA, USA). Higher-magnification images were acquired with a Nikon C2si laser scanning confocal microscope (Nikon, Amstelveen, The Netherlands) equipped with a 20× objective (Plan Apo, NA 0.75, Thermo Scientific, Waltham, MA, USA) at a resolution of 1024 × 1024 pixels, using a 2× average. Images of control and cKO brains were acquired with equal exposure times/laser and gain settings. Brightness and contrast of the overview images in Figure 3A were post hoc-adjusted in FIJI [85] (RRID: SCR_002285) for visualization purposes; adjustments were equally applied to control and cKO images.

### 2.5. Locomotor Phenotyping

Assessment of spontaneous movements was performed in an open field apparatus (Versamax, Omnitech, Columbus, OH, USA), simultaneously placing tamoxifen-treated *Atxn2l*-flox versus *Atxn2l*-flox/Cre mice at monthly intervals into 20 × 20 cm chambers, recording their activity using infra-red beams during 5 min trials, and interrogating predefined parameters for alterations with consistency over time, as previously described [61]. *Atxn2l*-flox/Cre-Tg animals were injected with TAM to induce the conditional KO (*Atxn2l*-cKO), in parallel with *Atxn2l*-flox mice that lacked Cre and served as controls (*Atxn2l*-flox/Cre-WT).

### 2.6. Tamoxifen Preparation and Treatment

A tamoxifen solution (20 mg/mL) was prepared from 200 mg of tamoxifen powder (Sigma-Aldrich #T5648, Burlington, MA, USA) and reconstituted in 10 mL of peanut oil (Sigma-Aldrich #P2144, USA). Tamoxifen was dissolved directly in peanut oil at room temperature with vertical rotation overnight and subsequent vortexing. Mice were weighed before the first injection, and the volume to be injected was calculated for a tamoxifen dose of 75 mg/kg body weight. Intraperitoneal tamoxifen injections were applied on five consecutive days, using 0.3 mL syringes and 30-gauge × 8 mm needles (Becton, Dickinson and Co. #324826, Franklin Lakes, NJ, USA).

### 2.7. Global Proteomics

Mice were euthanized through cervical dislocation, after which the brains were dissected, snap-frozen in liquid nitrogen, stored at −80 °C, and transported on dry ice. For proteomic analysis, eight left frontal cortex specimens (4 Atxn2l-cKO and 4 Atxn2l-flox/Cre-WT) were included. This sample number was chosen to allow for the exclusion of one potential mis-genotyped animal or outlier per group while still maintaining sufficient power to detect major effects. To uncover more subtle changes in future experiments, the use of a non-mosaic Cre driver line is advisable.

Tissues were homogenized in denaturing buffer using a FastPrep system (1 × 60 s, 4.5 m/s) in 800 µL of freshly prepared buffer (3 M guanidinium chloride, 10 mM tris(2-carboxyethyl)phosphine, 20 mM chloroacetamide, 100 mM Tris-HCl, pH 8.5). Lysates were heated to 95 °C for 10 min with shaking (1000 rpm, thermal mixer) and subsequently sonicated in a water bath for 10 min. After centrifugation, supernatants were transferred to low-binding 1.5 mL tubes (Eppendorf, Hamburg, Germany). Protein concentrations were quantified by BCA assay (Thermo Scientific, USA; kit no. 23252). Each sample (500 ng) was diluted in buffer containing 10% acetonitrile and 25 mM Tris-HCl, pH 8.0, to reach 1 M guanidinium chloride. Proteins were first digested with LysC (MS-grade, Roche, Basel, Switzerland; enzyme-to-protein ratio, 1:50) for 3.5 h at 37 °C, shaking at 800 rpm. The mixture was then diluted to 0.5 M guanidinium chloride and subjected to overnight tryptic digestion (Roche, 1:50 ratio, MS-grade) at 37 °C with agitation. Peptides were acidified with formic acid (final concentration, 2%) and loaded onto Evotip Pure tips (Evosep, Odense, Denmark) according to the manufacturer’s instructions.

Separation of peptides was performed on an Evosep One LC system using an Aurora Elite C18 column (15 cm × 75 µm ID, 1.7 µm beads; IonOpticks, Victoria, Australia) with the 20-samples-per-day method (Whisper Zoom 20-SPD). The LC was directly coupled to a timsTOF Ultra 2 mass spectrometer (Bruker Daltonics, Bremen, Germany) employing data-independent acquisition with the PASEF workflow. Spectral data were analyzed using Dia-NN (v2.0), searching against an in silico-predicted murine spectral library, with the “match between runs” option enabled. The mass spectrometry data have been deposited at the ProteomeXchange Consortium (http://proteomecentral.proteomexchange.org, accessed on 6 June 2024) via the PRIDE partner repository [86] with the data set identifier PXD064497.

### 2.8. Proteome Interrogation for Pathway Enrichments

The webserver of STRING version 12 (Search Tool for the Retrieval of Interacting Genes/Proteins) (https://string-db.org/, accessed on 6 June 2024) was employed to assess the proteome profile in an automated manner for any enrichments in protein–protein interactions, Gene Ontology (GO) terms, Reactome and KEGG pathways, subcellular localizations, and protein motifs [87]. In addition, to maximize bioinformatics insights, the three *Atxn2l*-flox/Cre-WT samples with the highest values and the three *Atxn2l*-cKO samples with the lowest values for ATXN2L abundance (reduction to 70% of controls) were compared for significant dysregulations in the proteome. The resulting list of proteins was visualized as a volcano plot and studied in STRING regarding ATXN2L-dependent interactomes.

### 2.9. Statistics and Graphical Presentation

Unpaired Student’s *t*-tests with Welch’s corrections were used to establish comparisons for continuous variables between homozygous *Atxn2l*-cKO and floxed WT animals. Mean values and variance as standard error of the means (SEM), as well as linear regression lines, were used for behavior data visualization. GraphPad (Version 10.4.1, for Windows, GraphPad Prism, Boston, MA, USA) was used for all statistical analyses and volcano plot generation. Significance was assumed at *p* < 0.05 and highlighted with asterisks: * *p* < 0.05.

## 3. Results

### 3.1. Generation of Conditional KO Mice via Floxing Exons 10–17 in the Atxn2l Gene

**Figure 1:**
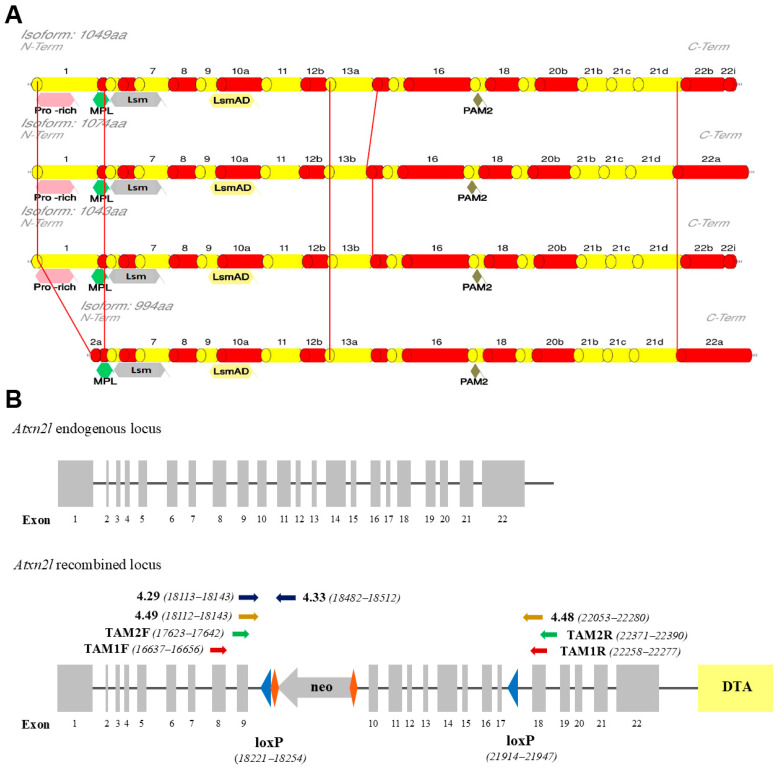
Structures of the murine Ataxin-2-like protein and gene. (**A**) Scheme of the ATXN2L protein domains (not depicted to scale), with the experimentally verified 1049 amino acid sequence above, and 3 predicted protein isoforms below. The reported Proline-rich, MPL-binding, Lsm, LsmAD, and PAM2 interaction motifs are shown in their positions relative to the coding exons, which are illustrated as red and yellow cylinders. Red lines highlight the differences between isoforms. (**B**) Scheme of the *Atxn2l* gene (not depicted to scale), with its endogenous allele above, versus its recombined allele below. Gray boxes: *Atxn2l* coding exons. Solid lines: intronic/intergenic regions. Gray arrow: neomycin (neo) as positive-selection cassette flanked by RoxP recombination sites (orange rhombuses) for in vivo excision. Blue triangles: loxP sites. Yellow box: diphtheria toxin fragment A (DTA) as a negative selection marker. Primer names and orientations are shown as colored arrows, with nucleotide numbers reflecting distance from the 126115977 end in genomic clone NC_000073.7.

To bypass the embryonic lethality of the constitutive ATXN2L-null genotype and to achieve ATXN2L absence selectively in neural tissue during adult life, we generated the first *Atxn2l*-cKO mouse strain. The initial bioinformatic characterization of the murine *Atxn2l* gene revealed 22 exons (Figure 1A), with ATG initiation codons in exons 1 and 2, stop codons in exon 22, translating into one experimentally validated protein with 1049 amino acids. This also provided protein isoforms containing 1074, 994, or 1043 amino acids (analyzing the database mRNA/protein entries in GenBank BC054483/UniProt Q7TQH0-1, GenBank AK155062/UniProt Q7TQH0-2, GenBank AK168745/NCBI NP_001334587, and isoform 4 derived from UniProt entry Q7TQH0-3). The variants showed N-terminal differences regarding the Proline-rich motif (PRM), while the N-terminal potential MPL–interaction region [72] was common to all (Figure 1A). The phylogenetically conserved sequences were always present, including the (i) Lsm domain involved in RNA binding, (ii) the LsmAD sequence (where a clathrin-mediated trans-Golgi signal, an ER exit signal, and a putative caspase cleavage site DxxD were reported), and (iii) the PABP-interaction motif PAM2 involved in mRNA turnover regulation. The ATXN2L variants showed differences at the C-terminus, where sequence homology exists with the Pat1 protein family [62].

To flank *Atxn2l* exons 10 and 17 with loxP sites, a targeting vector was generated in the mouse C57BL/6 genetic background, thus enabling the selective deletion of a 3.3 kb fragment containing the LsmAD sequence and the PAM2 motif, with a consequent frameshift that eliminates the C-terminal sequences. To drive efficient homologous recombination screens, this vector contained a neomycin-selection cassette with flanking RoxP sites between exons 9 and 10 as a positive selection marker, with murine isogenic *Atxn2l* genomic sequences comprising a short arm from exon 8 and a long arm extending beyond exon 22, together with diphtheria toxin fragment A as a negative selection marker (Figure 1B). An optimal screening methodology via polymerase chain reaction (PCR) was validated to (i) detect 5′ homologous recombination events; (ii) distinguish the wild-type, recombined, neo-excised, and Cre-excised alleles; and (iii) establish mouse genotypes (Figure 1B). After PCR identification of three ES cell clones with integration of the neomycin cassette in a heterozygous state and full sequencing into the surrounding locus for confirmation of genomic integrity, blastocyst injection generated three chimeric males with a chimerism rate above 50%. Three heterozygous floxed and (after crossbreeding with pan-Cre-deleter mice) three heterozygous constitutive mice were derived, with a second PCR screening performed to verify neo- and Cre-mediated excision events, followed by additional sequencing of the genomic locus. This approach generated 14 heterozygous floxed animals (7 males and 7 females) and 5 heterozygous constitutive KO mice (2 males, 3 females) in the F1 generation, which were interbred to generate F2 generations of the floxed conditional KO (*Atxn2l*-cKO) strain and the constitutive KO (*Atxn2l*-KO) strain of heterozygous breeders.

### 3.2. Crossbreeding with Constitutive Cre-Deleters Confirms Embryonic Lethality of Homozygous ATXN2L-Null Mice

Crossbreeding between heterozygous constitutive KO animals was performed to assess if the *Atxn2l* exon 10–17 deletion event causes embryonic lethality, similar to previously reported [83] constitutive *Atxn2l* exon 5–8 deletion, where the Lsm domain is missing (in addition to the absence of LsmAD/PAM2/Pat1 homology sequences in the current exon 10–17 strain). Among >100 collected offspring genotypes, no postnatal ATXN2L-null homozygote was observed (Table 1), in good agreement with the notion that embryonic lethality occurred again, despite the difference in targeted exons.

### 3.3. Crossbreeding with CamK2a-Dependent Cre/ERT2 Mice and Subsequent Tamoxifen Injection Generate Atxn2l-cKO in Adult Frontal Cortex Tissue

**Figure 2:**
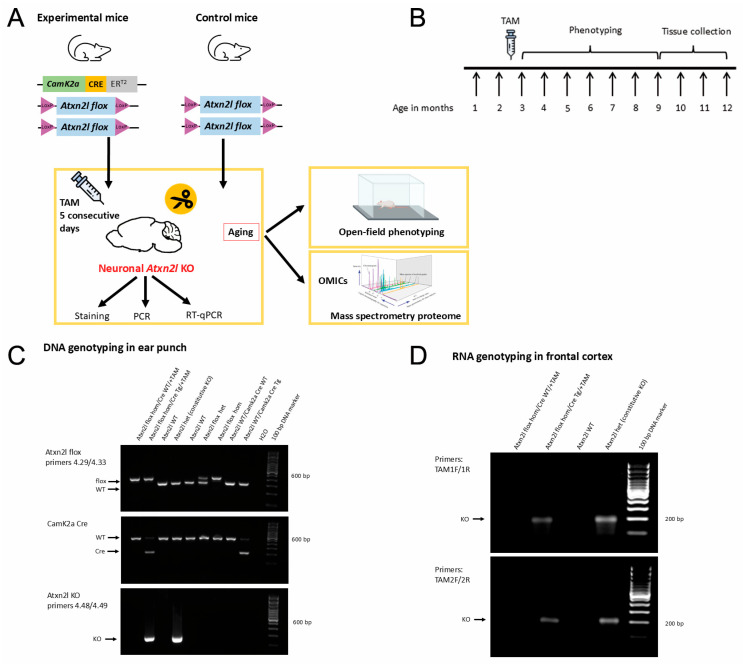
(**A**–**D**) Experimental design to cross *Atxn2l*-floxed mice with CamK2a-Cre/ER2 transgenic mice, administer tamoxifen, and control deletion success using genotypes at the DNA and RNA levels. (**A**) Planned crossbreeding and genotypes of *Atxn2l*-cKO and control mice. Mice were aged 2–3 months, injected with tamoxifen (TAM) over 5 consecutive days, subjected to locomotor phenotyping until the age of 9 months, and then had tissue collected for immunohistochemistry and proteomic analysis. (**B**) Experimental timeline. (**C**) DNA from ear punches of *Atxn2l*-flox hom/Cre-WT and *Atxn2l*-flox hom/Cre-Tg, both treated with TAM; different control mice were analyzed for the floxed locus, Cre presence, and successful *Atxn2l* deletion through efficient Cre expression. (**D**) RNA from frontal cortex tissue of *Atxn2l*-flox hom/Cre-WT and *Atxn2l*-flox hom/Cre-Tg, both treated with TAM; control mice were analyzed for successful *Atxn2l* deletion with SYBR Green technology using RT-qPCR. Primer details are provided in Table S1.

To selectively delete *Atxn2l* in CamK2a-positive neurons postnatally at the early adult age, homozygous *Atxn2l*-flox mice were obtained that differed regarding the heterozygous presence or absence of transgenic CamK2a-Cre/ERT2. Both were intraperitoneally administered tamoxifen over five consecutive days at ages of 2–3 months (Figure 2A). Their locomotor behavior was documented regularly until the age of 9 months, before tissues were dissected for further analyses (Figure 2B). PCR analyses at the DNA level in ear punches (Figure 2C) and at the RNA level in the frontal cortex (Figure 2D) confirmed the desired flox/Cre genotypes and demonstrated the successful *Atxn2l* deletion.

### 3.4. Atxn2l-cKO in Frontal Cortex Tissue Showed Mosaic Expression in CamK2a-Positive Neurons

**Figure 3:**
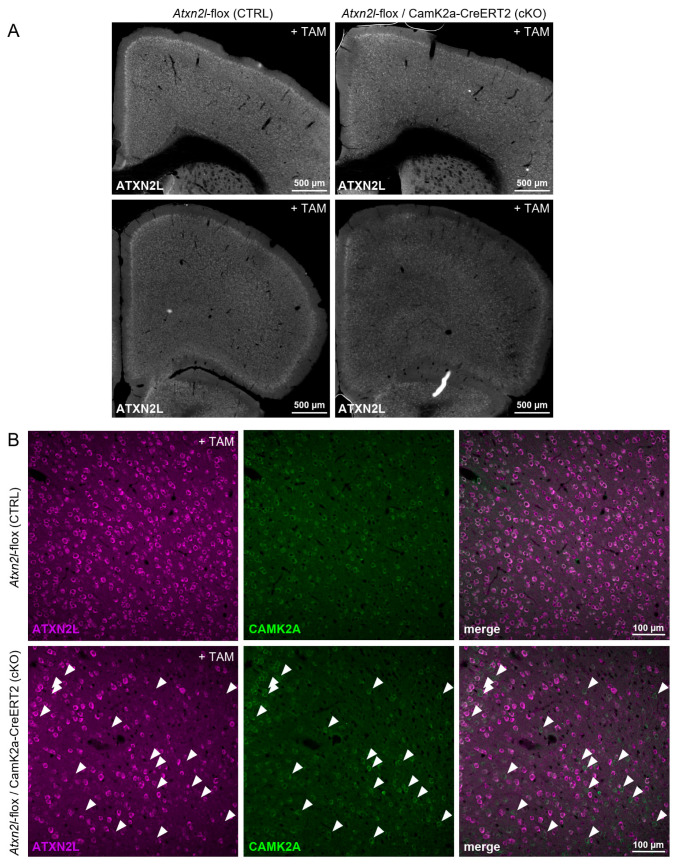
Mosaic depletion of ATXN2L protein in the frontal cortex of *Atxn2l*-flox/CamK2a-CreERT2 mice after TAM application (cKO). (**A**) Overview images of ATXN2L staining in the frontal cortex at different distances from the bregma. A reduction in the number of stained cells in a cKO mouse (right) compared with a control animal (left) is visible. (**B**) Higher magnification of a portion of the frontal cortex stained for both ATXN2L and CamK2a. In the control brain, virtually all neurons that are positive for CamK2a also express ATXN2L. ATXN2L staining that does not colocalize with CamK2a staining likely represents ATXN2L in interneurons and glial cells. In the cKO brain, a mosaic deletion of ATXN2L in CamK2a-expressing neurons can be observed. Filled arrows point to examples of CamK2a-positive cells that have lost ATXN2L presence.

To obtain a first impression of the cellular pattern of ATXN2L loss in the frontal cortex, immunofluorescence labeling for ATXN2L and CamK2a was employed. Serial brain sections of two cKO and two floxed WT mice were cut from the olfactory bulb to the hippocampus (one WT brain was discarded after postmortem genotyping). ATXN2L immunoreactivity was missing from many but not all CamK2a-positive neurons in the frontal cortex of the cKO mice (Figure 3) and hippocampus, demonstrating that either the transgenic CamK2a-Cre/ERT2 line or the tamoxifen dosage employed caused weak Cre expression, resulting in a mild chimeric deletion only in a part of the target cells. The observations in the brains investigated also indicate that ATXN2L deletion does not appear to cause widespread cell death or strong dedifferentiation of adult neurons even after >6 months.

### 3.5. CamK2a-Dependent Atxn2l-cKO Triggers Deficits in Spontaneous Horizontal Locomotion

**Figure 4:**
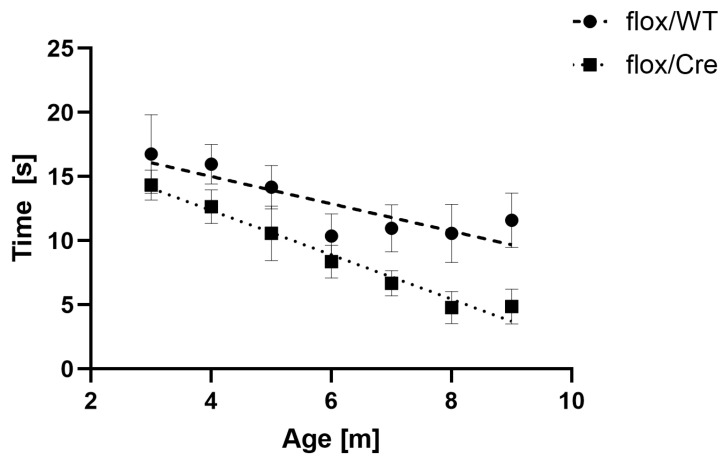
Phenotype progression in an open field paradigm, showing the parameter “ambulatory time” with mean values and variance as SEM, as well as linear regression lines. All animals studied were injected with tamoxifen, but only flox/Cre mice (*n* = 7–9) could produce a cKO in CamK2a+ neurons and have subsequent movement deficits, while *Atxn2l*-flox/Cre-Tg and *Atxn2l*-flox/Cre-WT mice (*n* = 5–6) are expected to recover normal movement activity over time.

To assess whether the absence of ATXN2L from the CamK2a+ neurons of the frontal cortex affects exploratory behavior, we quantified the spontaneous locomotor activity of mice after TAM injection in an open field paradigm after tamoxifen injection at monthly intervals from the ages of 3 to 9 months. The two weeks immediately after drug administration were exempted because animal handling with repeated intraperitoneal injections of tamoxifen dissolved in oil can transiently trigger discomfort or even peritonitis, resulting in a significant variability in behavior. The average spontaneous mobility parameters for cKO usually showed similar or lower values than floxed WT mice (except rest and stereotypy time), with consistent significant decreases for the parameter ambulatory time at 8 and 9 months (Figure 4). Overall, the data indicate that the chimeric dose reduction of ATXN2L in the CamK2a+ neurons of the frontal cortex was sufficient to alter curiosity and/or anxiety levels.

### 3.6. CamK2a-Dependent Atxn2l-cKO Mouse Frontal Cortex Proteomics Shows ATXN2L Protein Reduction to 75% and Dysregulation of the Alternative Splicing Pathway

**Figure 5:**
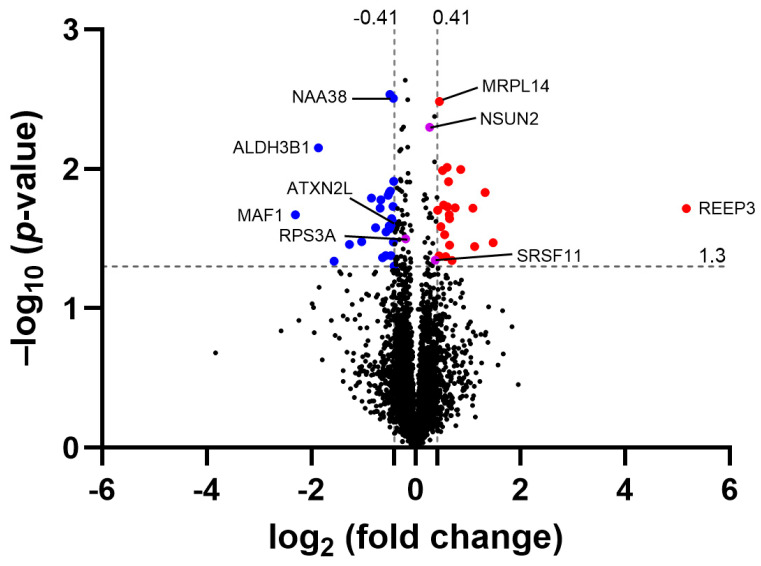
Volcano plot illustrating relevant dysregulations in the global proteome of the frontal cortexes of Atxn2l-cKO mice. A –log10 (*p*-value) of 1.3 corresponds to a *p*-value of 0.05 as the threshold for nominal significance; a log2 (fold-change) of −0.41 reflects the depletion of the ATXN2L protein. Factors with stronger downregulations are visualized as blue dots with gene symbols, with stronger upregulations in red and weaker dysregulations in purple.

To demonstrate the molecular consequences of ATXN2L-cKO in adult nervous tissue, samples from several important brain regions (olfactory bulb, frontal cortex, striatum, hippocampus, septum, tectum, and cerebellum) were analyzed using label-free mass spectrometry to quantify their global proteome profiles. In the frontal cortex, average ATXN2L protein abundance decreased to 75% in the four samples with nominal significance, after the tamoxifen-activated, Cre-mediated deletion events in CamK2a-positive neurons at ages >9 months (Figure 5, Table S1). This quantification result is credible, given that ATXN2L is physiologically present in these neurons, as well as in other neurons and glia, endothelial, and blood cells, where no deletion is expected in our experiment. The two proteins with significant ATXN2L dependence on murine embryonic fibroblasts—NUFIP2 as an RNA processing factor and SYNE2 as a bridge between the nuclear envelope and microtubules—did not display dysregulated levels (Table S1). In our complete analysis of four cKO samples, the strongest downregulation was observed for MAF1 as a repressor of RNA polymerases and the U6 snRNP, as well as the ribosomal translation apparatus, which is controlled by mTOR growth signaling [88]. Even more significant downregulation was observed for ALDH3B1 as a factor that protects medium-/long-chain fatty acids from lipid peroxidation. The strongest upregulation was documented for REEP3 as a bridge between the endoplasmic reticulum, with microtubules that ensure the nuclear envelope’s architecture [89,90].

Considering this mild decrease in ATXN2L levels upon CamK2a-dependent deletion, the downstream consequences would be too subtle for immunoblot analyses, and thus, only systematic bioinformatics surveys with alternate approaches were conducted.

STRING interaction and enrichment analysis revealed that protein dysregulations occurred with the strongest enrichment in dendrites (false discovery rate, FDR = 4.07 × 10^−8^); for cytoskeleton binding proteins (FDR = 4.36 × 10^−5^); and factors with rapid regulation by phosphorylation (FDR = 2.50 × 10^−7^), acetylation (FDR = 1.05 × 10^−5^), and alternative splicing (FDR = 9.61 × 10^−5^). The only enriched protein motifs were the pleckstrin homology domains (FDR = 0.04), which mediate binding to inositol lipids. As a single ATXN2L interactor with significantly dysregulated levels, NAA38 was reduced to 74% with nominal significance. NAA38 is a member of the snRNP family of Lsm domain-containing proteins and serves as an auxiliary component of the N-terminal acetyltransferase C (NatC) complex, which acts after ribosomal translation in the cytosol.

Other dysregulated RNA processing proteins with nominal significance included nuclear splicing factor SRSF11, RNA cytosine C(5)-methyltransferase NSUN2, and RPS3 and MRPL14 as ribosomal subunits in the cytosol and mitochondria, respectively; this was found in both the complete analysis of four cKO versus four floxed WT samples and in the enrichment analysis of three selected cKO mice with the lowest ATXN2L levels versus three floxed WT mice with the highest levels.

Upon assessing consistencies between the frontal cortex and hippocampal proteome profiles; normalization either among multiple brain regions; or normalization among these two regions with maximal ATXN2L depletion, an abundance of cytoplasmic ribonucleoprotein granule factors with nominal-significance dysregulations stood out (FDR = 0.0125). This involved decreases in ATXN2L and AGO3 versus increases in LSM3, EDC4, FXR2, NSUN2, LARP7, and SRSF11.

Overall, the mild CamK2a-dependent *Atxn2l*-cKO in the frontal cortex provided preliminary insights into the alterations in neural ribonucleoproteins and pathways that underlie the phenotypic deficits of spontaneous locomotion.

## 4. Discussion

Mutations of the less-abundant ATXN2 and the more-abundant ATXN2L have a preferential impact on neural tissues, with their distorted functions making causal contributions to the adult neurodegenerative diseases SCA2 and ALS. However, no mammalian model has been available where ATXN2L mutation effects can be studied in adult neurons. Furthermore, the primary protein–RNA interactions of ATXN2L in adult mammalian neurons have not been documented. Moreover, the exact physiological role of ATXN2L in RNA processing remains to be identified, both during growth periods, when its subcellular distribution shows perinuclear concentration, and in periods of cell damage, when its redistribution to stress granules suggests its involvement in quality control and repair.

Here, we generated the first *Atxn2l*-cKO mouse line via genetic ablation of exons 10–17 with a subsequent frameshift, interrupting ATXN2L N-terminal translation before the LsmAD and PAM2 domains and the C-terminal Pat1-homology region. Upon constitutive Cre-mediated deletion in homozygosity, embryonic lethality ensued, as previously reported for *Atxn2l* exon 5–8 deletion with a frameshift [83]. Upon conditional CamK2a-Cre/ERT2-mediated deletion in frontal cortex neurons via tamoxifen injection at the adult ages of 2–3 months, as well as subsequent aging to 9–12 months, cell death was not observed in CamK2a-positive neurons with completely absent ATXN2L. However, altered curiosity and/or anxiety were observed in response to the altered signaling of these neurons upon ATXN2L absence. Analyses of the global proteome of the frontal cortex (and hippocampus) from the *Atxn2l*-cKO mice confirmed a reduction in ATXN2L protein to 75% abundance, as well as weak downstream alterations in the alternative splicing pathway (e.g., SRSF11 and LARP7) and the cytoplasmic ribonucleoprotein granule composition. The ATXN2L protein is mainly cytoplasmic, and it appears to control the surveillance and triage of alternatively spliced isoforms, which need rapid turnover because of stress and stimulus. Our findings on the effects of ATXN2L Lsm and LsmAD are in excellent agreement with the well-established function of nuclear Sm domains for alternative splicing [91] and cytosolic Lsm1-7 rings for balancing mRNA translation versus turnover [92]. The rapid changes that occur between different alternative isoforms of any factor enable growth and compensate for stress; this ability appeared with the evolution of eukaryotic organisms [93], and indeed, Ataxin-2 family members also evolved in primitive eukaryotes. These organisms suffer from oxidative stress due to the endosymbiosis of mitochondria and chloroplasts. Failures in protective responses to radical oxygen species are a central cause of neurodegenerative processes, and physiological and pharmacological mechanisms of defense are being intensely explored, e.g., selenoproteins in the nervous system and the application of nano-selenium in treating mitochondrial problems [94,95].

Our observation of chimeric and mosaic deletions upon CamK2a-driven Cre/ERT2 expression and tamoxifen administration has been reported previously [96,97,98,99]. Similarly, altered curiosity-driven exploration and/or anxiety is an expected feature upon frontal cortex dysfunction [100,101,102]. Furthermore, an impact on alternative splicing was previously shown for mutations in TDP-43, FMR1, NUFIP2, and G3BP2 [103,104,105,106], as they are ATXN2/ATXN2L ribonucleoprotein interactors and stress granule components [82]. Thus, the results for the new *Atxn2l*-cKO mouse align with previous work.

The main limitations of this study derive from the choice of the CamK2a promoter to selectively drive weak Cre expression in the frontal cortex and hippocampus. Given that Cre transgene expression is weak when controlled by the CamK2a promoter, ATXN2L depletion was mild in the current project, and the downstream proteome dysregulations were too subtle for validation using independent techniques such as immunoblots and quantitative RT-qPCR. Therefore, to maximize the effect sizes of downstream proteome dysregulations, the next round of *Atxn2l*-cKO experiments could be driven by a strong promoter with specificity for forebrain and hippocampus projection neurons, such as the NEX-Cre transgene [107]. While affecting the frontal cortex and hippocampus is unlikely to restrict the survival of mouse mutants—thus providing a safe opportunity to assess the viability of ATXN2L depletion in adult neurons—future experiments should also target motor neurons, cerebellar neurons, and brainstem neurons, which are more critical and correspond to the neural circuits affected by ALS and SCA2. All current mass spectrometry findings must be regarded as preliminary, given that no single result achieved actual significance, and the findings varied in dependence on tamoxifen efficacy in different animals, diverse brain regions, and normalization approaches. However, it was illustrative to identify both NAA38 and LSM3 among the nominally significant dysregulations, given that ATXN2L and both factors mentioned are members of the Lsm protein family and that LSM12 was previously reported as an ATXN2L interactor [82]. The mainly nuclear ribonucleoproteins NSUN2 and SRSF11 were also consistently dysregulated, indicating that ATXN2L depletion leads to adaptations in nuclear RNA processing. NSUN2 acts with ALYREF and the ATXN2 interactor YBX1 to bind m5C-mRNAs and modify their nuclear export [108,109]. SRSF11 (also known as p54 or SRp54) always resides in the spliceosome [110,111], acting as a constitutive splicing factor, but it associates with U2AF65, unlike other members of the SR family [112]; furthermore, its specific functions include the splicing of small introns [113] and neuronal microexons [114].

Overall, this project generated the first mammalian conditional *Atxn2l* deletion strain, providing a proof of concept demonstrating that it constitutes a useful tool for future analyses of ATXN2L physiological functions in adult brains, which have selective importance for neuronal responses to stress and stimuli.

## 5. Conclusions

This study generated the first mouse strain with conditional deletion of ATXN2L, providing evidence that (i) the complete absence of ATXN2L is incompatible with embryonic development and (ii) the chimeric removal of ATXN2L protein levels from half of the CamK2a-positive frontal cortex neurons triggers reduced spontaneous locomotion and dysregulated protein levels in the alternative splicing pathway in adult mice.

## Figures and Tables

**Table 1:** Offspring counts. After crossing with constitutive Cre-deleter mice, Atxn2l^+/−^ intercrosses produce offspring with postnatal genotype distribution that confirms embryonic lethality upon homozygous exon 10–17 deletion.

Observed/Expected Number of Live Born Mice with Indicated Genotype				
	+/+	+/−	−/−	Number of Offspring
Live born female	**33**/27	**39**/54	**0**/27	**72**/108
Live born male	**20**/27	**26**/54	**0**/27	**46**/108
Live born total	**53**/54	**65**/108	**0**/54	**118**/216

## Data Availability

All proteome data were deposited in PRIDE with accession number PXD064497.

## Supplementary Materials

The following supporting information can be downloaded from https://www.mdpi.com/article/10.3390/cells14191532/s1, Table S1. Primers used for genotyping; Table S2. Proteome frontal cortex; Table S3. STRING analysis from proteome frontal cortex.

## Abbreviations

The following abbreviations are used in this manuscript:

AGO3	Argonaute RISC Catalytic Component 3
ALDH3B1	Medium-Chain Fatty Aldehyde Dehydrogenase
ALS	Amyotrophic Lateral Sclerosis
ALYREF	Aly/REF Export Factor
ATG	Autophagy-Related Gene
ATX	Ataxin
ATXN2	Ataxin-2
ATXN2L	Ataxin-2-Like
AU-rich	Adenosin-Uridine Rich
BCA	Bicinchoninic Acid
bp	Base Pair
CAG	Cytosine–Adenine–Guanine Trinucleotide Repeat
CamK2a	Calcium/Calmodulin-Dependent Protein Kinase II Alpha
cKO	Conditional Knock-Out
Cre	Cyclization Recombination Enzyme
DEAD	Asp-Glu-Ala-Asp (DEAD-box helicase family)
DIA	Data-Independent Acquisition
DNA	Deoxyribo-Nucleic Acid
DTA	Diphtheria Toxin Fragment A
DxxD	Aspartate–Any Amino Acid–Any Amino Acid–Aspartate
EDC4	Enhancer of mRNA Decapping 4
EGF	Epidermal Growth Factor
ER	Endoplasmic Reticulum
ERT2	Tamoxifen-Inducible Estrogen Receptor Domain, Improved Version
ES	Embryonic Stem Cell
FDR	False Discovery Rate
FEBS	Federation of European Biochemical Societies
FIJI	FIJI Is Just ImageJ
Floxed	Sequence Flanked by Two loxP Sites
FMRP	Fragile X Mental Retardation Protein
FMR1	Fragile X Messenger Ribonucleoprotein 1
FXR2	Fragile X Mental Retardation, Autosomal Homolog 2
GO	Gene Ontology
G3BP2	GTPase Activating Protein (SH3 Domain) Binding Protein 2
hATXN2	Human Ataxin-2
het	Heterozygous
hom	Homozygous
HSC	Hematopoietic Stem Cell
kb	Kilo-Base
KEGG	Kyoto Encyclopedia of Genes and Genomes
KO	Knock-Out
LARP7	La Ribonucleoprotein 7, Transcription Regulator, Binds U6 snRNA
LC	Liquid Chromatography
LED	Light Emitting Diode
LoxP	Locus of X-Over (Crossing-Over) in Bacteriophage P1
Lsm	Like-Sm Domain
LsmAD	Like-Sm-Associated Domain
LSM3	LSM3 Homolog, U6 Small Nuclear RNA-Associated
MAF1	MAF1 Homolog, Negative Regulator of RNA Polymerase III
MPL	Myelo-Proliferative Leukaemia Protein
mRNA	Messenger Ribo-Nucleic Acid
MRPL14	Large Ribosomal Subunit Protein UL14m
MS	Mass Spectrometry
mTOR	Mechanistic Target of Rapamycin
mTORC1	Mechanistic Target of Rapamycin-Complex-Associated Protein 1
m5C	5-methyl-Cytosine
NAA38	N-Alpha-Acetyltransferase 38, NatC Auxiliary Subunit
NGS	Normal Goat Serum
NSUN2	NOP2/Sun RNA Methyltransferase 2
NUFIP2	Nuclear Fragile X Mental Retardation Protein Interacting Protein 2
oligo(U)	Several (Uridine Bases)
OR	Odds Ratio
PABP	Poly(A)-Binding Protein
PAM2	Poly(A)-Binding protein association Motif type 2
PASEF	Parallel Accumulation Serial Fragmentation
Pat1	Yeast Factor for Protection of mRNA 3′-UTRs from Trimming
P-bodies	Processing Bodies in the Cytosol, Where RNA Is Degraded
Pbp1	Poly(A)-Binding Protein 1 in Yeast
PBS	Phosphate-Buffered Saline
PCR	Polymerase Chain Reaction
Poly(A)	many (Adenine bases)
polyQ	Many Glutamine Amino Acids
PRM	Proline-Rich Motif
REEP3	Receptor Expression-Enhancing Protein 3
RNA	Ribo-Nucleic Acid
RNP	Ribo-Nucleoprotein
RoxP	loxP-Analogous Site, Recognized by Phage Integrase Dre
RPS3	Small Ribosomal Subunit Protein US3
RRM	RNA Recognition Motif
rRNA	Ribosomal Ribo-Nucleic Acid
RT	Reverse Transcription
SCA2	Spinocerebellar Ataxia type 2
SD	Standard Deviation
SEM	Standard Error of the Mean
SG	Stress Granule
siRNA	Small Interfering RNA
SN	Substantia Nigra
SNP	Single Nucleotide Polymorphism
snRNA	Small Nuclear Ribo-Nucleic Acid
snRNP	Small Nuclear Ribo-Nucleic-Acid Binding Protein
SR	Serine/Arginine-Rich Protein, Family of Splicing Factors
SRSF11	Serine and Arginine Rich Splicing Factor 11
SYNE2	Spectrin Repeat Containing Nuclear Envelope Protein 2
TAM	Tamoxifen
TDP-43	TAR DNA-Binding Protein-43
Tg	Transgenic
tRNA	Transfer Ribo-Nucleic Acid
UPR	Unfolded Protein Response
UTR	Untranslated Region of mRNA
U2AF65	U2 Small Nuclear RNA Auxiliary Factor 2
WB	Western Blot
WT	Wild-Type
YBX1	Y-Box-Binding Protein 1, CCAAT-Binding Transcription Factor I
ZFE	Zentrale Forschungseinrichtung (Central Animal Facility)
